# Chemosensitive Properties of Electrochemically Synthesized Poly-3-Thienylboronic Acid: Conductometric Detection of Glucose and Other Diol-Containing Compounds under Electrical Affinity Control

**DOI:** 10.3390/polym16131938

**Published:** 2024-07-07

**Authors:** Yulia Efremenko, Vladimir M. Mirsky

**Affiliations:** Nanobiotechnology Department, Institute of Biotechnology, Brandenburg University of Technology Cottbus-Senftenberg, 01968 Senftenberg, Germany

**Keywords:** glucose sensor, saccharide sensor, chemosensitive polymer, poly-3-thienylboronic acid, conductometric sensor, glucose biosensor

## Abstract

Due to the presence of the boronic acid moieties, poly-3-thienylboronic acid has an affinity for saccharides and other diol-containing compounds. Thin films of this novel chemosensitive polymer were synthesized electrochemically on the gold surface. The adhesion of the polymer was enhanced by the deposition of a monomolecular layer of thiophenol. The technology was used to fabricate conductometric sensors for glucose and other diol-containing compounds. Simultaneous two- and four-electrode conductivity measurements were performed. The chemical sensitivity to sorbitol, fructose, glucose, and ethylene glycol was studied at different pH and electrode potentials, and the corresponding binding constants were obtained. Depending on the electrode potential, the reciprocal values of the binding constants of glucose to poly-3-thienylboronic acid at neutral pH are in the range of 0.2 mM–1.0 mM. The affinity for glucose has been studied in buffer solutions and in solutions containing the major components of human blood. It was shown that the presence of human serum albumin increases the affinity of poly-3-thienylboronic acid for diol-containing compounds.

## 1. Introduction

The high importance of glucose analyses is the main motivation for the search for chemical sensors for diol-containing compounds. Glucose is the central carbohydrate in human physiology. It exists in two isomeric forms, but only one isomer, D-glucose, is involved in human metabolism through the process of glycogen degradation. Glucose levels are regulated by insulin, and its failure causes diabetes mellitus. Blood glucose control is essential to prevent the increase and progression of diabetic complications and to improve the effectiveness of therapy. The World Health Organization (WHO) predicts that the number of adults with diabetes will reach nearly 700 million by 2045 [1]. The global prevalence of diabetes and the consequent need to assess glycemic status has led to the establishment and development of sensors for glucose detection.

The first glucose sensors were based on the use of glucose oxidase as a selective recognition element. The enzyme was combined with sensors for the co-substrate (oxygen) or by-product (hydrogen peroxide) of the enzymatic reaction of glucose oxidation. Subsequently, new generations of glucose biosensors have been successfully implemented and commercialized, including glucose test strips with electrochemical or optical readout or based on the direct electrical measurement of the rate of the enzymatic reaction [2,3,4,5,6]. This can be performed either invasively or non-invasively [7]. Today, this segment dominates the market of commercially available sensors, accounting for 85% of global biosensor production.

Although enzyme-based electrochemical glucose biosensors have gained widespread market and scientific acceptance, these devices have significant drawbacks. The principal drawback of these sensors is their instability caused by the biological nature of the enzyme. Enzymes are sensitive to a number of factors, including freezing or temperatures above ~50 °C; the presence of heavy metals, oxidizing agents, or detergents; a too-basic or -acidic pH level; very low or very high ionic strength; etc. [8,9]. This limits the range of applications for enzymatic sensors and necessitates the implementation of specific storage and transportation protocols. Such sensors are unsuitable for use in devices that require continuous control (e.g., in bioreactors) with regular cleaning and sterilization. This has led to an intensive search for enzyme-free analytical techniques for the detection of glucose.

The first attempt at enzymeless glucose detection was conducted with polarography on a mercury electrode [10]. This work was subsequently followed by electrochemical oxidation of glucose using platinum [11,12,13] and gold electrodes [14,15]. In recent years, there has been a trend to develop electrochemical glucose sensors based on electrocatalytically active materials, such as immobilized gold nanoparticles [16] and carbon nanotubes [17]. All of these techniques are based on measuring the rate of the electrochemical oxidation of glucose. However, attempts to use these technologies in real conditions are complicated by the adsorption of albumin or other compounds on the electrode surface, which blocks or interferes with the electrochemical reaction. These problems have led to the search for affinity sensors that are less susceptible to interference.

The design of affinity sensors requires the presence of a receptor for the specific binding of the analyte. However, the number of functional groups that can be used to specifically recognize glucose is very limited. The search for such affinity groups is complicated by the competition between glucose and water to form hydrogen bonds with receptors. In particular, the molar concentration of water is approximately four orders of magnitude greater than that of glucose. Only a few molecules or functional groups have been found to satisfy these requirements. The first is the group of natural compounds known as lectins. The affinity of these molecules for monosaccharides can be further increased by introducing additional subunits or subsites into their structure [18]. However, they also belong to proteins or glycoproteins and therefore have the same stability problems as the enzymes.

A number of synthetic receptors that have been proposed to bind carbohydrates in the aqueous environments include calixarenes, cholic acid, porphyrins, cyclodextrins, and boronic acid moieties [19]. However, most of these synthetic receptors, except the boronic acid moiety, have very low binding constants (1–100 L/mol) and can hardly be applied in chemical sensing for the detection of millimolar or sub-millimolar concentrations. This makes boronic acid the most promising synthetic receptor for saccharide chemical sensing [20,21]. The binding chemistry of this receptor has been the subject of detailed study in [22]. The application of sensors and the development of molecular probes based on the boronic acid moiety are reviewed in detail in [23,24]. In aqueous solutions, boronic acid interacts strongly and reversibly with 1,2- or 1,3-diols to form an anionic cyclic boronate ester. The optimal binding depends on a combination of factors, such as the pKa values of the boronic acid and diol [25], pH [26], and solution composition [27].

The boronic acid moiety can be introduced into conducting polymers to make them sensitive to saccharides, including glucose [28,29,30,31]. This is usually achieved with the pre-synthetic modification of the monomers and subsequent synthesis. The synthesized copolymer of aniline and 3-aminophenylboronic acid was reported in [32] and showed sensitivity to various saccharides; however, the detection limit for glucose was only 45 mM. Better results for the potentiometric detection of glucose (4–6 mM) were obtained with the polymerization of only 3-aminophenylboronic acid [26]. The binding of glucose can also be performed with the use of gold nanoparticles modified with mercaptophenylboronic acid [33]. Recently, we have reported the electrochemical synthesis of a new polymer containing the boronic acid moiety–poly-3-thienylboronic acid (PThBA) [34].

Here, we describe the fabrication of a conductometric sensor based on this polymer and characterize its chemosensitive properties in buffer solutions as well as in a solution containing the major components of human blood. The study of the affinity properties of PThBA has shown that in contrast to its monomer characterized in [35], the polymer exhibits a pronounced sensitivity to glucose at neutral pH and in the presence of a high concentration of albumin. Another important feature of this new polymer material is its electrical conductivity and electrochemical activity. As is the case with other affinity sensors based on electrochemically active materials [36,37], the affinity properties of this polymer can be modulated by the electrode potential. This makes PThBA a promising material for applications in both simple conductometric sensors for the detection of glucose or other diol-containing compounds and in electrically controlled chemotransistors [37]. The latter approach permits the realization of electrically accelerated sensor recovery [36] or the formation of a virtual sensor array based on the single receptor unit [38].

## 2. Experimental

### 2.1. Materials

3-Thienylboronic acid, boron trifluoride diethyl etherate (BFEE), 2,6-di-*tert*-butylpyridine (DTBP), thiophenol, D-sorbitol, D-fructose, D-(+)-glucose, ethylene glycol, human serum albumin (HSA), lithium chloride, sodium carbonate, and sodium bicarbonate were obtained from Sigma-Aldrich (Darmstadt, Germany). Acetone, ethanol, and acetonitrile (ACN), were purchased from Th. Geyer (Germany). Sodium hydrogen phosphate, sodium dihydrogen phosphate, and sodium chloride were obtained from Roth (Roth, Germany). The pH of the buffer solutions was adjusted with 1 M solutions of NaOH or HCl from Roth (Roth, Germany). All aqueous solutions were prepared with deionized water and further purified using the EGLA-Classic system (Hermsdorf, Germany). Purchased chemicals were used as received. Measurements were performed at room temperature.

**Figure 1:**
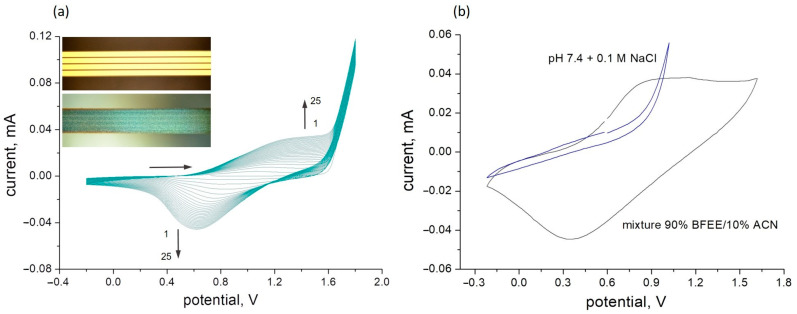
The electrochemical deposition of PThBA on the bare gold electrodes with a thiophenol sublayer (**a**) and the cyclic voltammetry of the deposited PThBA film in aqueous (grey curve) and in organic (blue curve) electrolytes (**b**). Polymerization was performed from 50 mM 3-thienylboronic acid dissolved in 90% boron trifluoride diethyl etherate and 10% acetonitrile (*v*/*v*) for 25 cycles. In the inset, light microscopy images of the electrodes before (**top**—bare gold surface) and after (**bottom**—emerald colored polymer film) the electrochemical deposition of PThBA are shown. Aqueous electrolyte: 100 mM phosphate buffer pH 7.4. The organic electrolyte: 90% boron trifluoride diethyl etherate and 10% acetonitrile (*v*/*v*). Scan rate: 100 mV/s.

The sensor chip was fabricated by Fraunhofer IZM (Munich, Germany) using a photolithographic lift-off technique on a 0.5 mm thick glass wafer, with 150 nm thick gold structures produced by sputtering. Ti/W sublayer was used to enhance the adhesion of the gold on a glass wafer. The sensor comprised six linear electrodes: two outer and four inner electrodes. The inner measuring electrodes, which were used for resistance measurements, were in the form of four parallel strips comprising two outer and two inner electrodes of 35 µm width, separated by 8 µm gaps (Figure 1, inset).

### 2.2. Instrumentation and Procedures

Prior to electropolymerization, the sensor chips were cleaned with acetone, ethanol, water, and piranha solution (mixture of 30% H_2_O_2_:concentrated H_2_SO_4_, 1:3 (*v*/*v*). Caution: this solution is highly reactive with most organic materials and must be handled with extreme care); rinsed thoroughly with deionized water; and then dried. A standard three-electrode configuration was used for polymer deposition. PThBA films were polymerized from a 0.05 M solution of 3-thienylboronic acid in a mixture of 90% BFEE and 10% acetonitrile (*v*/*v*) containing 0.05 M DTBP with potential cycling from −0.2 V to +1.8 V vs. an Ag/AgCl reference electrode (Metrohm, Herisau, Switzerland) filled with 2 M LiCl in ethanol with the same solution in the salt bridge. Platinum wire with a geometrical area of ~10 mm^2^ was used as the auxiliary electrode. To improve the adhesion of the PThBA film to the gold surface, the sensor chip was pre-coated with a self-assembled monolayer of thiophenol from a 10 mM solution in ethanol for 30 min. followed by rinsing with ethanol and drying. The electrochemical synthesis was performed using the Autolab PGSTAT-12 General Purpose Electrochemical System (USA) with Nova 2.1.4 software.

In situ conductance measurements were performed using the simultaneous two- and four-point measurements (s24) [39] at the defined potential relative to the reference electrode. This technique enables the resistance of the polymer and contacts to be measured simultaneously. This provides an internal integrity control of the conductometric sensor, the details of which are described in [36]. Briefly, 10 mV pulses of opposite polarity with 3.5 s duration were applied to the outer electrodes using the programmed Keithley K2400 voltage source meter (USA), which also measures the current through the outer electrodes. The voltage drop across the two inner electrodes was measured using a high impedance voltmeter, Keithley 617 (USA). The software to control the measurement system was based on HP-VEE tools. The Ag/AgCl (sat.) reference electrode (Metrohm, Herisau, Switzerland) with a double salt bridge (sat. KCl) was used. The potential values in the conductance measurements are presented with respect to this reference electrode.

The characterization of conductive polymer films was carried out in 0.1 M phosphate and carbonate buffers containing 0.1 M NaCl. Buffer solutions were prepared by dissolving calculated amounts of (i) potassium hydrogen phosphate and potassium dihydrogen phosphate or (ii) sodium carbonate and sodium bicarbonate in water.

## 3. Results and Discussion

### 3.1. Electrochemical Deposition of Thin Films of PThBA

The deposition of the chemosensitive polythiophene derivative onto the gold measurement electrodes was performed using electropolymerization according to a technique first reported in [34] and then continued in [40]. It was proposed that electrochemically synthesized PThBA has a linear structure. A more detailed discussion of the PThBA structure, in particular of this polymer synthesized by enzymatically catalyzed chemical synthesis, is presented in [41]. In the previous work [34], it was demonstrated that the transfer of the polymer films, which were electrochemically synthesized on ITO electrodes, to the aqueous phase leads to the detachment of the polymer and the formation of free-standing polymer films of ~100 nm thickness. The presence of sulfur atoms in the polymer structure provided the rationale for the use of gold to enhance polymer adhesion. The cyclic voltammograms observed during the deposition on the gold surface were similar to those obtained for the deposition on ITO glass slides, which were performed under the identical conditions and from the same electrolyte [34]. The polymerization led to the formation of smooth, homogeneous films of PThBA. The strong lateral polymer growth resulted in the complete filling of the gaps between the gold strips (Figure 1, inset). The number of cycles determines the thickness of the film.

**Figure 2:**
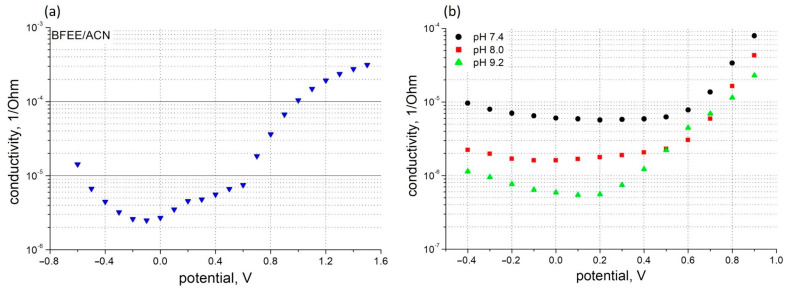
Potential dependence of PThBA conductance measured in four-electrode configuration in organic electrolyte (**a**) consisting of 90% boron trifluoride diethyl etherate and 10% acetonitrile, *v*/*v*, and in aqueous electrolytes (**b**) at pH 7.4 (circles), pH 8.0 (squares), and pH 9.2 (triangles).

However, initial experiments have shown that the adhesion of the formed PThBA film to the electrode surface remains insufficient for the fabrication of sensors for applications in aqueous media. Despite the gold support, an exposure to an aqueous solution for as little as one hour caused the film to peel off. This effect can be exploited for the synthesis of free-standing polymer films of defined thickness. However, this represents a great obstacle to the fabrication of conductometric sensors. In order to enhance the adhesion of PThBA to the substrate, the technology previously developed for the deposition of other conducting polymers [42,43] was used. Precoating the gold surface with a monolayer of thiophenol resulted in strong adhesion of the formed PThBA film and high stability of the formed PThBA/gold structure in aqueous environments. This can be explained by the inclusion of this compound as the first monomer into the formed polymer chain and by surface hydrophobization, which prevents water penetration to the gold surface. The cyclic voltammogram of the electrochemical polymerization of ThBA on the gold surface coated with this sublayer was identical to that obtained for the polymerization of ThBA on the bare gold surface (Figure 2). This indicates that the deposited adhesion sublayer does not contribute to the electron transfer resistance.

### 3.2. The Electrochemical Activity and Conductivity of PThBA

The electrochemical activity of the deposited PThBA films in aqueous solutions was not observed (Figure 1). This is consistent with the earlier reported behavior of polythiophene [42], whose electrochemical activity is also blocked in aqueous solutions. This observation provides an indirect indication of the hydrophobicity of the PThBA film, which prevents the penetration of hydrophilic ions from aqueous electrolytes. In organic electrolytes comprising 90% boron trifluoride diethyl etherate and 10% acetonitrile (*v*/*v*), the reversible oxidation and reduction were observed, with potential values of the oxidation and reduction peaks of +0.8 V and +0.35 V, respectively. The potential gap is slightly lower than that for PThBA deposited on ITO [34]. The difference can be attributed to either a better contact of the polymer with the gold due to the application of the adhesive sublayer or to the different organic electrolytes used.

The characterization of the electrical conductivity of PThBA films was performed in aqueous solutions. The application of simultaneous two- and four-point measurement techniques showed that for the film deposition with a thiophenol adhesive sublayer, no instances of film detachment were observed during the entire course of our investigation. To characterize the polymer conductivity under different conditions in the presence and the absence of analytes, the values measured by the four-point measurement technique were used. The dependence of the PThBA conductivity on the applied potential is shown in Figure 2. A high increase in the film resistance was observed, with a slope of 10-fold increase per 220–350 mV with a tendency for the slope to decrease at alkaline pH. The potential value at which the resistance increases is ~+0.2 V at pH 9.2 and between ~+0.55 and ~+0.6 V for more neutral pH values. These results suggest the presence of at least two redox states of the polymer with different specific conductivities. A small increase in cathodic potentials may indicate the existence of a third state with a conductivity higher than the midpoint of the potential scale. An increase in the conductance at higher electrode potentials was also observed for polythiophene [42], but in this case, the dependence clearly indicated at least three redox states. The essential difference between the conductivities of polythiophene and PThBA, which can be attributed to the influence of the boronic acid moiety, was observed in the most reduced polymer state: the conductivity of polythiophene decreases with decreasing potential (Figure 2 from [43]), whereas PThBA shows a tendency to increase conductivity (Figure 2).

**Figure 3:**
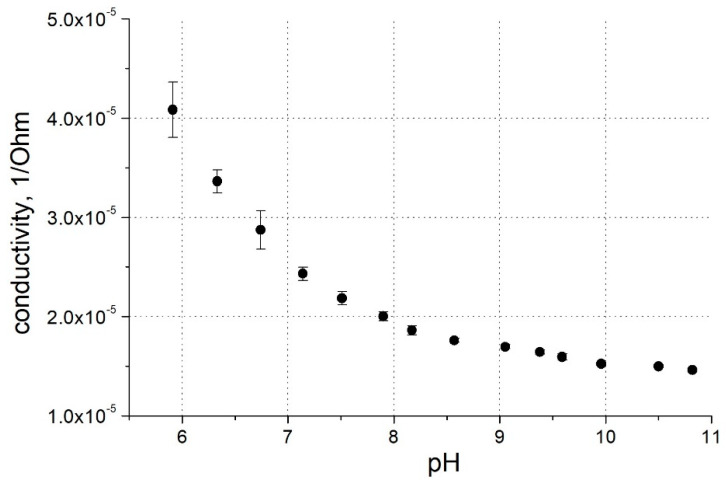
pH dependence of PThBA conductance at 0.0 V potential. Electrolytes: 100 mM phosphate buffer for pH 6.0–pH 8.5, 100 mM carbonate buffer for pH 9.0–11.0.

The incorporation of the boronic acid moiety into the polythiophene backbone leads to a pH effect on the polymer conductivity (Figure 2b and Figure 3). At alkaline pH, the deprotonation of this functional group led to a decrease in PThBA conductivity. A quantitative analysis of the pH effect on the polymer requires a study of the surface electrostatics and the influence of the pH shift near the polymer surface; such a study was performed with PThBA nanoparticles [41]. From the conductance dependence (Figure 3), it can be concluded that the pKa value of PThBA is less than 7. The monomer of this polymer has been reported to have a pKa of 8.1 [35]. This indicates a pKa shift towards the acidic direction due to polymerization. A similar effect was observed for PThBA nanoparticles, where a pKa value of ~8.6 was obtained [41]. However, the direct comparison of the pKa values of the electrochemically prepared PThBA film and the chemically synthesized PThBA nanoparticles may be affected by the influence of the polymerization technology and consequently by the resulting polymer structure.

### 3.3. The Chemosensitive Properties of PThBA Films

**Figure 4:**
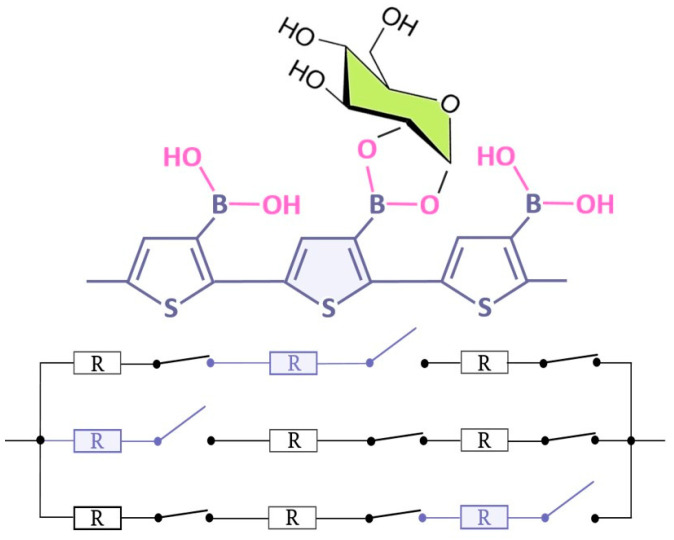
The formal electrical model describing the influence of the analyte on the resistance of a thin film of PThBA. Assuming independent analyte binding to the binding sites of different polymer chains and energetic homogeneity of the binding sites, the conductance versus concentration curves can be considered as proportional to the Langmuir adsorption isotherms.

The main motivation for studying PThBA is its potential application in chemical sensors. One can imagine that the binding of an analyte (saccharide or other diol-containing compound) to a boronic acid moiety of a monomer of the PThBA polymer chain leads to a change in the chain resistance at this binding site. The following sections demonstrate that this change is an increase in the chain resistance. If we consider the polymer film as a set of parallel polymer chains, we can use a simple electrical model (Figure 4). The transfer of charge between the polymer chains is not considered. In this case, the change in conductance is directly proportional to the number of chains in which at least one monomer is bound to the analyte. It is assumed that all binding sites of the polymer exhibit identical properties and that there is no mutual influence between the binding sites. The low value of the relative conductance change at the maximum saccharide effect indicates the existence of another pathway that is independent of the saccharide concentration; this may be caused by an electrical leakage in the polymer film. If the resistance of the whole polymer chain in the absence of occupied binding sites is proportional to the film thickness while the resistance of the polymer chains with at least one occupied binding site is infinite, we may conclude that the relative conductance change obeys a Langmuir adsorption isotherm. Normalization to the maximum conductance allows us to minimize the influence of variations in the film thickness and consequently the scatter of the data.

**Figure 5:**
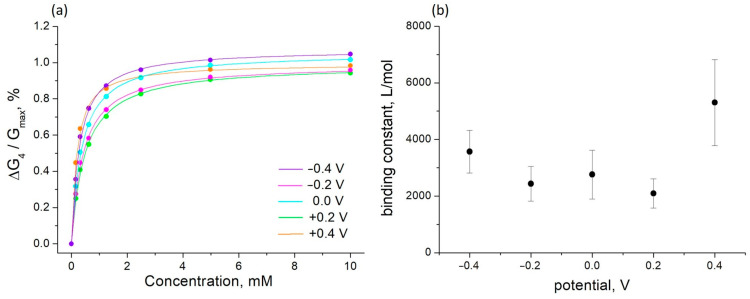
The influence of sorbitol on the conductance of PThBA ((**a**), circles) and its fitting by the Langmuir adsorption isotherm ((**a**), continuous lines) at different electrode potentials and the dependence of the extracted values of the affinity constants on the electrode potential (**b**). The standard deviation value in (**a**) is comparable to the size of the circles, and the error bars in (**b**) correspond to the Langmuir isotherm fitting of the concentration dependence.

The experimental data on the changes in the conductance of the PThBA film in the presence of sorbitol are presented in Figure 5. Modifying the redox state of the PThBA by varying the electrode potential led to some changes in these curves. In the anodic region, the potential scale for such measurements is limited by the value of ~+0.5 V. At higher potentials, irreversible damage to the PThBA film is observed.

The concentration dependence obeys the Langmuir adsorption isotherm well (Figure 5a, continuous lines). This can be considered as proof of the postulates of independence and homogeneity of the binding sites. The values of the binding constants obtained from this fit are shown in Figure 5b. It can be observed that the binding constant increases several times at potentials above +0.2 V. This effect, indicating the formation of a PThBA redox state with a higher affinity for saccharides, may be related to a strong increase in the PThBA conductance observed at anodic potentials (Figure 2b). At the potential of +0.2 V, the binding constant is ~2.5 times lower than at +0.4 V. This dependence allows for the receptor properties to be tuned to achieve maximum sensor efficiency over the required concentration range or to speed up sensor recovery. This allows us to consider PThBA as a promising receptor material for electrochemical chemotransistors with electrically controlled affinity [44] for the accelerated sensor recovery [36] or virtual sensor arrays [38].

According to our data on the study of the binding of different saccharides to ThBA, the highest affinity was observed for sorbitol. Similar results have been obtained for the binding of saccharides to other boronic acid-containing compounds [45]. It was shown that the selectivity of phenylboronic acid towards monosaccharides has the following sequence: fructose ˃ galactose ˃ glucose [46]. To explain the selectivity series, it must be taken into account that saccharides in an aqueous solution exist in a number of anomeric forms with different affinities to the boronic acid moiety. The furanose form is the most suitable for the formation of boronate esters [47]. Therefore, the measured affinity constants refer to some averaged values that are influenced by the ratio of anomers under certain conditions and in particular by the percentage of the furanose anomer.

**Figure 6:**
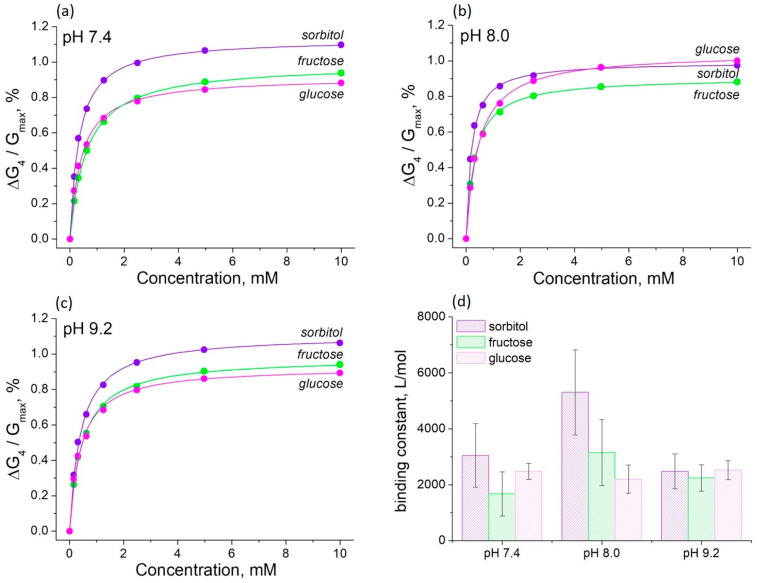
The influence of saccharides on the PThBA conductance at different pH at the electrode potential of +0.4 V at pH values of 7.4 (**a**), 8.0 (**b**), and 9.2 (**c**) and the affinity constants for the binding of sorbitol, fructose, and glucose at these pH values (**d**). The affinity constants were obtained by fitting the concentration dependence by the Langmuir isotherms (continuous lines).

The effects of sorbitol, fructose, and glucose on the conductance of PThBA films are shown in Figure 6. The polymerization of ThBA has a dramatic effect on the affinity of the boronic acid moiety for saccharides, resulting in the suppression of selectivity (Figure 6). The measurements were performed at an electrode potential of +0.4 V, at which the strongest binding of sorbitol was observed. Considering the favorable influence of alkaline pH [48,49] and the practical importance of measurements at neutral pH [50], the experiments were performed at pH 7.4, 8.0, and 9.2. In all cases, the concentration dependence obeys the Langmuir adsorption isotherm (Figure 6, continuous curves).

The binding constants for all the saccharides studied (including glucose) are in the range of 1700–5800 L/mol. The binding of saccharides to ThBA has shown a strong pH effect, e.g., changing in pH from 7.4 to 9.2 results in a ~13-fold increase in the binding constant [35]. The binding properties of PThBA show almost no pH dependence (Figure 6d), except for sorbitol. However, even for this compound, the overall pH effect was less than 3-fold. The difference in the affinity for all the saccharides studied is also much smaller. Note that PThBA also has a high affinity for glucose, whereas this value is very low for the monomers containing a single boronic acid moiety [45,51].

Although the data obtained do not allow an explicit interpretation, two possible mechanisms can be suggested. The first one is a shift in the electron density in the vicinity of the boronic group due to polymerization that leads to an increase in the affinity of this group. In this case, however, it is unclear why a similar effect has never been observed as a result of a chemical modification of the monomers. A further mechanism is based on the binding of one saccharide molecule to two boronic acid moieties, which results in a doubling of the total binding energy, while the value of the binding constant is squared. A similar mechanism was considered to explain the condensation of PThBA nanoparticles in the presence of saccharides [41]. In Langmuir adsorption isotherms, it can be represented by a sum of two hyperbolic terms. Although fitting with such a model does result in a smaller deviation, this may only be a mathematical result because of the introduction of two additional fitting parameters. Furthermore, it is possible that some inhomogeneity of binding sites in polymers may be caused by a difference in the local environment of receptor groups. Finally, we consider multiple binding to be the most probable explanation; however, we cannot exclude other mechanisms.

### 3.4. The Detection of Glucose in the Presence of Albumin

**Figure 7:**
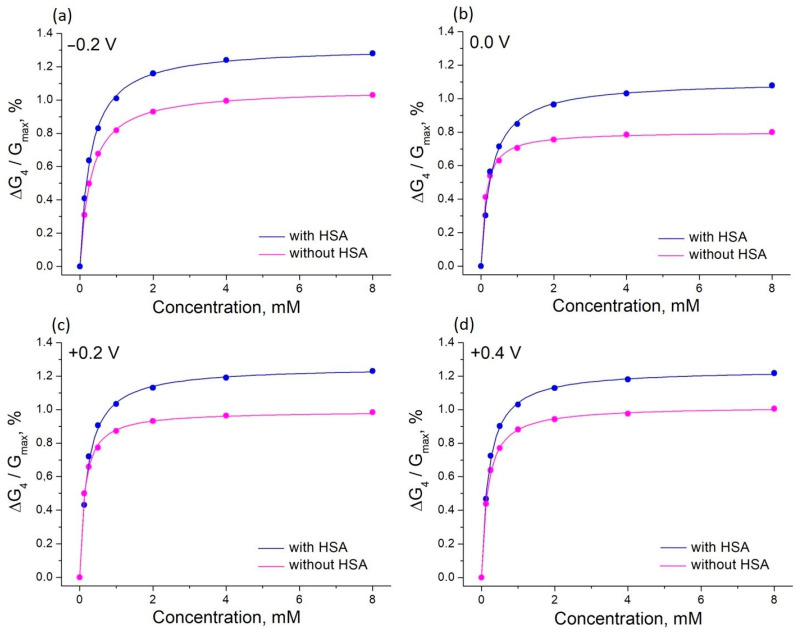
The effect of glucose on the relative conductance of PThBA in 1 mM phosphate buffer solution containing 100 mM NaCl, 27 mM sodium bicarbonate, and 42.5 mg/mL HSA (pH 7.4) and in the same electrolyte without HSA measured at four different potentials. The curves were fitted with Langmuir isotherms, the obtained affinity constants are 3400–5210 L/mol (without HSA) and 6300–8070 L/mol (with HSA).

Many attempts to develop chemical sensors for use in blood have failed: the sensors worked well in simple buffer or salt solutions but were severely affected by high concentrations of albumin. This was the reason for studying the effect of glucose on the PThBA conductance in a solution containing human serum albumin and the major inorganic ions of human blood at concentrations equivalent to those found in human blood. Such solutions are routinely used to test and calibrate clinical sensors and sensor kits for glucose. The binding isotherms and their fitting with Langmuir adsorption isotherms are shown in Figure 7. 

**Figure 8:**
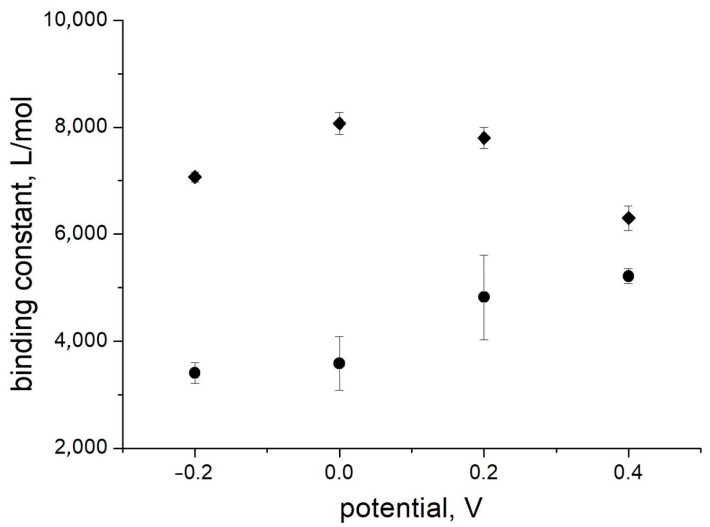
The influence of the electrode potential on the affinity of glucose for PThBA in 1 mM phosphate buffer solution containing 100 mM NaCl, 27 mM sodium bicarbonate, and 42.5 mg/mL HSA (pH 7.4) and in the same solution without albumin. The error bars correspond to the data fitting error in Figure 7.

Surprisingly, the values obtained for the binding constants are higher than for the same solutions without albumin. The influence of the electrode potential on the binding constant was also different, with a maximum in the potential range 0.0–0.1 V (Figure 8). Therefore, the presence of a high concentration of albumin does not lead to any suppression of the sensor signal; on the contrary, a positive influence on the affinity is observed. The signal changes were observed in the clinically relevant concentration range. The influence of albumin on the sensor affinity is not important for measurements in blood when the albumin concentration is relatively constant. Therefore, an application of PThBA in chemical sensors for the direct measurement of glucose in blood can be proposed.

Two mechanisms can be considered for the influence of different compounds on the observed affinity of the boronic acid moiety to saccharides. The first effect is the direct influence on the binding energy due to the formation of a complex that also includes the interfering compound. The second mechanism is the influence of an interfering compound on the equilibrium between different conformations. Saccharides exist in aqueous solutions as a mixture of anomers. In particular, glucose exhibits an anomeric equilibrium between the pyranose and furanose forms and a negligible amount of the hydrated acyclic aldehyde. Although the pyranose forms predominate in the free glucose equilibrium, only α-glucofuranose has a furanose ring with a cis-1,2-diol on the opposite sides of the ring. For this reason, the furanose anomeric form of glucose is most suitable to bind boronic acid. To calculate the true values of the binding constants, it is necessary to take into account the concentration of α-glucofuranose, i.e., the conformation that is bound [20,28]. Therefore, the values obtained when using the total ligand concentration are, in fact, the apparent values of the binding constants, whereas the true values are higher. The introduction of any compound capable of binding one of these conformations leads to a shift in the equilibrium and a corresponding change in the concentrations of all the other anomeric forms.

Saccharides may interact with certain physiological compounds. For example, some saccharides are known to be reduced by phosphate to form enols [52]. Saccharides also interact with proteins: the non-enzymatic glycation has been studied in detail [53,54], in particular the interaction between glucose and albumin. This is a spontaneous reaction in which glucose molecules bind to free amino groups (N-terminal residues) of the protein to form glycosylated albumin, which is stable under physiological conditions [55]. Human serum albumin (HSA) is highly susceptible to glycation due to the presence of lysine, arginine, and cysteine residues with high nucleophilic properties. Lysine is considered to be the major site of HSA glycation, accounting for 30% of the total protein glycation by glucose [56]. The proportion of glycated albumin in the blood of healthy individuals is in the range of 1–20%, whereas in people with diabetes, it can be 2–3 times higher [53,57]. Although it is difficult to imagine that glucose being associated with albumin can bind boronic acid more strongly than free glucose, this process may promote the formation of boronate esters by shifting the equilibrium towards the furanose anomer.

**Figure 9:**
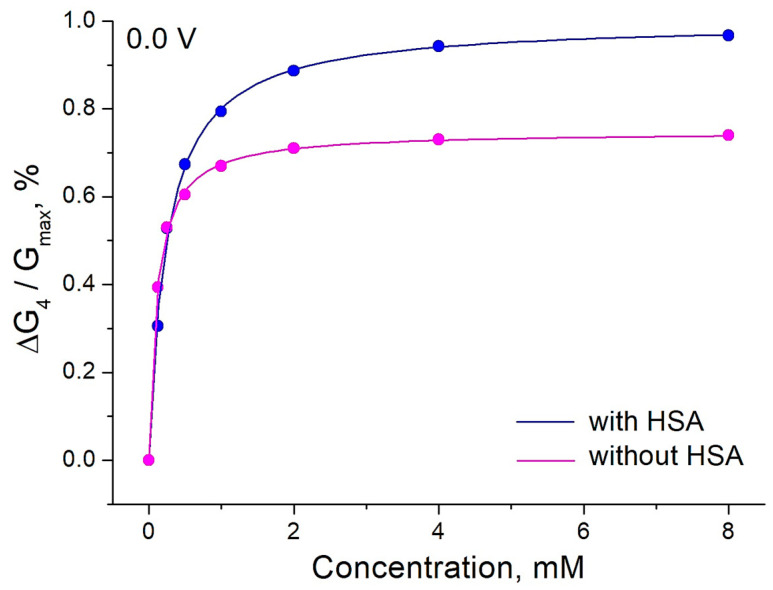
The effect of ethylene glycol on the relative conductance of PThBA in a 1 mM phosphate buffer solution containing 100 mM NaCl, 27 mM sodium bicarbonate, and 42.5 mg/mL HSA (pH 7.4) and in the same electrolyte without HSA. The data were fitted with Langmuir isotherms. The values obtained for the affinity constants are 4040 L/mol (without HSA) and 8560 L/mol (with HSA).

This hypothesis was tested by studying the influence of albumin on the binding of ethylene glycol to PThBA. The experiments were performed in two solutions: in the solution containing physiological concentrations of human serum albumin and the main inorganic salts as well as in the same solution without albumin (Figure 9). In an aqueous solution, the central bond of ethylene glycol (OCCO) is predominantly present in the gauche conformation (~80%), in which intermolecular interactions influence the conformation of the terminal OH groups and the dihedral angle CCOH can assume a wide range of values [58]. This allows for the OH groups of this form of ethylene glycol to bind to the boronic acid moiety. Ethylene glycol as the simplest diol has only one pair of OH groups, which precludes the possibility to bind two boronic acid moieties. Therefore, any influence on the ethylene glycol solution can lead to not more than a 20% increase in the form that can bind the receptor, resulting in a maximum 20% increase in the apparent binding constant. However, the effect of albumin on PThBA is explained by a 2-fold increase by varying the concentrations of conformers with different binding properties. Therefore, the effect of albumin can be explained by the first mechanism—an involvement in the formation of the boronate ester. A more detailed investigation of the mechanism of albumin on the glucose to PThBA binding requires a special study with the intensive use of spectroscopic techniques (FTIR, NMR) and computer modelling, which is beyond the scope of this work.

**Figure 10:**
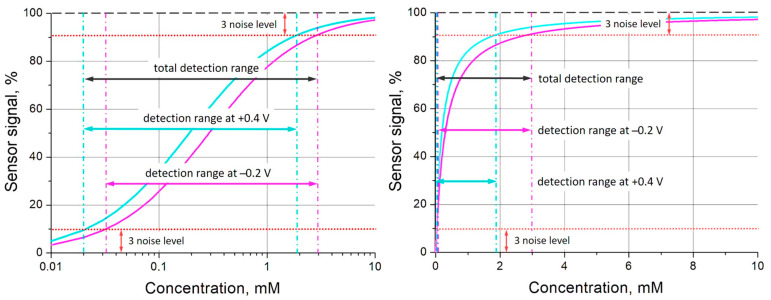
An extension of the detection range for glucose detection by the combination of measurements at two different potentials: +0.4 V (cyan curve, binding constant 5200 L/mole) and −0.2 V (magenta curve, binding constant 3400 L/mole). The detection ranges were determined for a 3% level of noise. The binding isotherms are shown in logarithmic (**left**) and linear (**right**) concentration scales.

Analytical performance of affinity sensors is completely defined by the affinity constant of the receptor used and signal-to-noise ratio. There were sophisticated attempts to go beyond the limit of classical Langmuir adsorption isotherms using competitive or cooperative effects [59,60], but they can be realized only for some defined analytes and conditions. The use of redox-active materials as receptors allows one to apply an electrical control of the receptor affinity, whereby each redox state of the receptor material can exhibit different binding properties [37]. This study has demonstrated that PThBA also belongs to the materials with an electrically controlled affinity (Figure 5b, Figure 7, and Figure 8). Let us consider an example of the application of this material to extend the detection range of glucose using the values of binding constants from the data in Figure 8, represented by circles. The Langmuir isotherms were calculated for the values of the binding constant at the potential of +0.4 V (5200 L/mole) and −0.2 V (3400 L/mole), with a noise level of 3%, and are shown in Figure 10. According to the IUPAC guidelines [61], the detection range was determined as the sensor signal corresponding to the 3.3 times noise level or to such deviation from the saturation level. The data demonstrate that the measurements at two different potentials lead to a significant extension of the detection range.

## 4. Conclusions

In this study, we described a new conducting polymer—PThBA. This polymer is a derivative of polythiophene. The new polymer was obtained with the electrochemical polymerization of 3-thienylboronic acid. The boronic acid group introduced into the thiophene has an affinity for diols. Polymerization of this compound led to the formation of a polymer with a conductive polythiophene backbone, while the incorporated boronic groups provided chemosensitivity to diols. The polymer was deposited onto gold electrodes. An anchoring monomolecular layer of thiophenol was used to achieve a strong adhesion of PThBA to the surface and to obtain highly stable structures that can be used in conductometric chemical sensors. It was observed that in contrast to polythiophene, which exhibits three redox states with different conductivities for each state and no pH dependence of conductivity, the potential dependence of PThBA did not show a clear switch between redox states. However, a strong pH influence on the polymer conductivity was observed. The binding of saccharides led to a decrease in the polymer conductivity. Such effects were measured for different saccharides, including sorbitol, fructose, and glucose. The experiments were performed at different pH and electrode potentials. In contrast to its monomeric form, PThBA exhibits a broad specificity for different saccharides. The binding constants were found to be higher than those observed for the monomer. In particular, the effect of pH was relatively minor, and a strong binding was observed even at neutral pH. This fact makes the new chemosensitive material highly promising for potential analytical applications. The polymer can also be prepared in the form of nanoparticles, which also exhibit an affinity for saccharides [41].

This optimism was reinforced by an analysis of the effects of glucose. It is well known that the boronic acid moiety has a relatively low affinity for glucose [45,46]. The monomeric form of the polymer studied is not an exception: it was not possible to obtain a quantifiable calorimetric response due to the interaction of glucose with TBA [35]. However, for the polymeric form of this material (PThBA), glucose effects are well measurable and strongly pronounced even at physiological pH. This last unexpected but also promising effect is the effect of a high concentration of albumin: an increase in the affinity of PThBA for glucose was observed under such conditions. The values of the reciprocal binding constants are in the range of 0.1–1 mM, allowing for effective use of this sensor in the concentration range of a few millimoles, which is the most important for many biotechnological and medical applications.

PThBA is a redox-active polymer that can exist in different redox states. The transition between these redox states can be controlled by applying an electrical potential. This leads to the possibility of the electrical control of the affinity properties of PThBA. Such effects have been demonstrated and can be exploited in electrochemical chemotransistors [62] for fast sensor recovery [36] or the realization of virtual sensor arrays [38]. Other possibilities include an exploitation of the optical properties of this polymer [34] and the development of more sophisticated sensing devices that combine optical and electrical transduction.

A further improvement in the analytical performance of PThBA can be achieved by implementing molecularly imprinted polymerization. This technology is well compatible with electrochemical polymerization [63,64,65]. A significant improvement in binding selectivity can be expected with the electrochemical synthesis of PThBA in the presence of the analyte.

It has been demonstrated that the new polymer, PThBA, is a promising chemosensitive material for applications in various chemical sensors for the detection of saccharides and other diol-containing compounds.

## Data Availability

The original contributions presented in the study are included in the article, further inquiries can be directed to the corresponding author.
